# Warfarin overdose successfully treated using prophylactic vitamin K infusion without severe coagulopathy: A case report

**DOI:** 10.1002/ams2.70036

**Published:** 2025-01-09

**Authors:** Ryosuke Omoto, Yutaka Umemura, Yuki Kokubu, Takeyuki Kiguchi, Satoshi Fujimi

**Affiliations:** ^1^ Division of Trauma and Surgical Critical Care Osaka General Medical Center Osaka Japan

**Keywords:** INR, overdose, vitamin K, warfarin

## Abstract

**Background:**

Warfarin, a vitamin K antagonist, is widely used for preventing and treating thromboembolic diseases. While guidelines exist for managing elevated prothrombin time‐international normalized ratio (INR) in patients on warfarin, the treatment for warfarin overdose in these patients is yet to be standardized.

**Case Presentation:**

A 41‐year‐old woman ingested 230 mg of warfarin with suicidal intent, along with other medications. Initially unconscious, her INR was 1.0, and laboratory results were normal. Prophylactic continuous menaquinone‐4, vitamin K2, injections were administered before the INR increased. After stopping vitamin K2 72 h later, her INR rose to 1.8, but she recovered without severe coagulopathy or bleeding, despite a high initial warfarin concentration.

**Conclusion:**

This is the first case of warfarin overdose managed with prophylactic vitamin K2 injections before INR elevation, successfully preventing severe complications. Prophylactic vitamin K infusion may be a practical approach for warfarin overdose treatment in non‐dependent patients.

## Graphical abstract

Prophylactic vitamin K2 infusion successfully managed a warfarin overdose in a non‐warfarin‐dependent patient, preventing severe coagulopathy and hemorrhage despite the high initial warfarin concentration. This case suggests that early VK administration may be effective in avoiding complications in similar overdose scenarios.

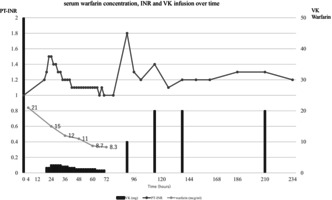

## BACKGROUND

Warfarin has been widely prescribed as an oral anticoagulant for over half a century for the treatment and prevention of various coagulopathic and thromboembolic diseases.^1^ Warfarin functions by antagonizing vitamin K (VK), thereby inhibiting the biosynthesis of VK‐dependent clotting factors (factors II, VII, IX, and X) as well as proteins C and S in the liver, resulting in anticoagulant and antithrombotic effects.^1^ Treatment with warfarin necessitates frequent laboratory monitoring using the prothrombin time‐international normalized ratio (INR) because of its extensive interpatient variability and narrow therapeutic range.^2^

Bleeding is a major adverse complication of warfarin therapy. The risk of bleeding associated with warfarin administration increases with greater INR, particularly when the INR exceeds 4.5.^3^ VK is a commonly used warfarin reversal agent in patients with supratherapeutic INR. The 2012 CHEST guidelines established the management of INR beyond the therapeutic range in warfarin‐dependent patients, where it is recommended that patients with INR <10 without bleeding should not receive VK.^4^ However, existing guidelines do not sufficiently describe the treatment of acute warfarin overdose. Consequently, a standardized treatment protocol for acute warfarin overdose has not been established and there are few reported cases.^5^, ^6^, ^7^, ^8^, ^9^

## CASE PRESENTATION

A 41‐year‐old woman ingested 230 mg of warfarin, 32.5 mg of clonazepam, 75 mg of chlorpromazine, and 6 g of acetaminophen with suicidal intent. Found unconscious by a neighbor approximately 3 h later, she was brought to the emergency department (ED). Her medical history included dissociative disorder and post‐traumatic stress disorder, but she was not on warfarin; her husband, however, took 6.25 mg daily due to mechanical heart valves.

Upon ED's arrival, she was drowsy but able to communicate; consciousness level was E1V1M5 on the Glasgow Coma Scale. Her vital signs on admission were as follows: blood pressure, 110/64 mmHg; heart rate, 58 beats per minute; respiratory rate, 19 breaths/min; blood oxygen saturation, 100% (supplemental oxygen, 5 L/min); and body temperature, 36.8°C. Laboratory test results were within normal limits; the initial INR was 1.0. CT scans showed no hemorrhage but indicated drug mass in the gastrointestinal tract. Gastric lavage and activated charcoal were administered. Prophylactic 50 mg intravenous menaquinone‐4, vitamin K2 (VK2), was given and serial INR was monitored every 2 h in the intensive care unit (ICU).

**FIGURE 1:**
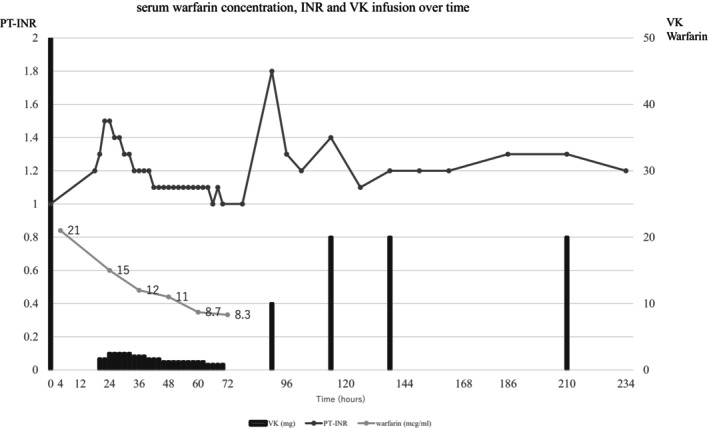
Figure shows the trends of serum warfarin concentration, INR, and VK infusion.

On day 2, the patient's INR increased to 1.3; Subsequently, continuous infusion of intravenous VK2 (40 mg/day) was initiated. On the same day, the dose of VK2 was increased to 60 mg/day, gradually tapered off, and discontinued on day 4. The patient was discharged from the ICU without further elevation of INR, and INR measurements were transitioned every 6 h. On day 5, the patient's INR increased to 1.8, and she was administered 10 mg of VK2. VK2 doses of 20 mg were administered on days 6 and 7 with INR 1.4 and 1.2, respectively. On day 10, as the INR was still 1.3 without improvement, VK doses of 20 mg were administered. Serum warfarin concentrations were measured every 12 h for a total of six measurements until day 3. The peak concentration of serum warfarin was 21 μg/mL at 4 h and gradually decreased to 8.3 μg/mL at 72 h (Figure 1). On day 8, whole‐body CT revealed no bleeding complications. The patient required psychiatric intervention for suicidal thoughts and was discharged on day 34 without complications of severe coagulopathy or hemorrhage.

## DISCUSSION

Warfarin is widely used as an oral anticoagulant; however, acute warfarin toxicity often occurs due to its extensive interpatient variability and narrow therapeutic range. Management of acute warfarin toxicity is complicated because of its pharmacokinetics.^2^ Warfarin is rapidly and completely absorbed in the gastrointestinal tract, and a maximal serum concentration is observed 90 min after ingestion. Its half‐life ranges between 29 and 45 h, but the coagulant effect of warfarin is prolonged in cases of warfarin overdose, with a duration of action of approximately 5 days.^5^, ^6^

A standardized protocol for managing acute warfarin overdose in patients who are warfarin‐independent is yet to be established. There is controversy regarding whether VK should be administered immediately to prevent INR elevation and potential bleeding events or withheld until a specific INR threshold or onset of symptoms. In several previous cases, prophylactic administration of VK before INR elevation was refrained.^5^, ^6^, ^7^ Notably, early administration of VK may have provided false reassurance and prevented close INR follow‐up, resulting in INR rebound. Berling et al. reported a 50‐year‐old man who overdosed on warfarin and initially presented with an INR of 2.5, received 6 mg of intravenous VK, but was re‐hospitalized the next day due to epistaxis and an INR rebound to 8.5.^8^ Another case involved a 15‐year‐old who ingested 350 mg warfarin with an initial INR of 1.1. He received 5 mg VK, but his INR increased to 5 3 days later, necessitating a fresh frozen plasma (FFP) transfusion.^9^ These cases highlight the importance of vigilant INR monitoring and the potential for missed INR spikes due to VK's short half‐life compared to warfarin's long half‐life.

In many previous cases, physicians did not always follow the CHEST guidelines; rather, they typically administered VK before the INR exceeded 10 even if there was no bleeding tendency, possibly owing to concerns regarding the continued absorption of warfarin and further inhibition of the vitamin K cycle. Watson et al. suggested an INR ≥4.5 as the threshold for VK administration, as seen in a 15‐year‐old girl who ingested 100–200 mg warfarin.^7^ Katherine et al. reported that VK was administered to a patient on warfarin overdose when the INR increased from 2.8 to 5.6.^5^ Levine et al. summarized 23 overdose cases where most patients received VK after their INR exceeded 3.^6^

However, several cases of bleeding or hematoma because of the delay in administering VK have been reported.^6^, ^7^ In Watson's reported case, the INR peaked at 6.67 at hour 85 resulting in duodenal hematoma.^7^ Levine et al. reported two patients with bleeding complications: one experienced retroperitoneal hematoma, and the other experienced delayed epistaxis and bleeding from the vocal cords.^6^

In our case, the prophylactic administration of VK combined with close INR monitoring effectively prevented severe complications following a warfarin overdose. In particular, the continuous infusion of VK appeared to provide a stable therapeutic effect, with the INR maintained at approximately 1.0–1.1. After discontinuation of the continuous infusion, the INR increased to 1.8, reflecting the shorter duration of VK's effects compared to warfarin's prolonged half‐life. Although no prior evidence demonstrates the benefits of continuous administration, these findings suggest the potential effectiveness of continuous VK infusion as a therapeutic strategy in managing acute warfarin overdose, as well as the importance of close INR monitoring.

The measurement of serum warfarin concentrations was outsourced, taking a month to obtain results, which limited our clinical utility. Quicker results could help predict INR rebound after VK administration and determine how long strict INR monitoring should be continued. In our case, continuous intravenous VK was administered for 72 h to prevent INR rebound, effectively controlling INR during this period. However, after stopping VK, the patient's INR rose to 1.8, with serum warfarin concentration reaching 8.3 μg/mL. Watson et al. found similar trends, suggesting close monitoring might be needed until day 10.^7^

To the best of our knowledge, this is the first case report detailing the use of continuous infusion of prophylactic VK before INR elevation. However, this is a single case, and further accumulation of similar cases is necessary to validate the effectiveness and safety of this strategy, but in patients without warfarin dependence experiencing warfarin overdose, prophylactic administration of sufficient doses of VK before INR elevation may be considered.

## CONCLUSION

The management of warfarin overdose remains uncertain regarding immediate VK administration versus guideline‐recommended withholding until a specific INR or symptoms appear. In this case, the efficacy and safety of prophylactic VK administration for patients without thromboembolism risk are highlighted. This approach may be considered for similar cases, emphasizing the need for further research to establish standardized treatment protocols.

## CONFLICT OF INTEREST STATEMENT

The authors declare no conflict of interest for this article.

## ETHICS STATEMENT

Approval of research protocol: N/A.

Informed consent: Informed consent for publication was obtained from the patient.

Registry and the registration no. of the study/trial: N/A.

Animal studies: N/A.

## Data Availability

Data sharing is not applicable to this article as no new data were created or analyzed in this study.
